# Susceptibility Vessel Sign and Intravenous Alteplase in Stroke Patients Treated with Thrombectomy: A Secondary Analysis of the SWIFT DIRECT Trial

**DOI:** 10.1007/s00062-025-01501-y

**Published:** 2025-02-20

**Authors:** Morin Beyeler, Roman Rohner, Petra Ijäs, Omer F. Eker, Christophe Cognard, Romain Bourcier, Igor Sibon, Margaux Lefebvre, Sébastien Richard, Arturo Consoli, Solène Moulin, Marielle Ernst, Marc Ribo, Charlotte Barbier, Omid Nikoubashman, David S. Liebeskind, Martina B. Goeldlin, Eike I. Piechowiak, Lukas Bütikofer, Jan Gralla, Urs Fischer, Johannes Kaesmacher, the SWIFT DIRECT investigators

**Affiliations:** 1 Department of Neurology, Inselspital, Bern University Hospital, University of Bern, Rosenbühlgasse 25, 3010 Bern, Switzerland; 2 Graduate School for Health Sciences, University of Bern, Bern, Switzerland; 3 Institute for Diagnostic and Interventional Neuroradiology, Inselspital, Bern University Hospital, University of Bern, Rosenbühlgasse 25, 3010 Bern, Switzerland; 4 Department of Neurology, Helsinki University Hospital and University of Helsinki, Helsinki, Finland; 5 Department of Vascular Neurology, Hospices Civils de Lyon, Lyon, France; 6 Department of Diagnostic and Therapeutic Neuroradiology, Centre Hospitalier Universitaire de Toulouse, Toulouse, France; 7 Department of Diagnostic and Therapeutic Neuroradiology, University Hospital of Nantes, L’institut du thorax, Nantes, Pays de la Loire, France; 8 Department of Diagnostic and Therapeutic Neuroradiology, CHU de Bordeaux, Bordeaux, France; 9 Department of Radiology, CHU Rouen, Rouen, France; 10 Department of Neuroradiology, CHRU-Nancy, Université de Lorraine, Nancy, France; 11 Department of Stroke and Diagnostic and Interventional Neuroradiology, Foch Hospital, Suresnes, France; 12 Department of Neurology, CHU Reims, Reims, France; 13 Department of Diagnostic and Interventional Neuroradiology, University Medical Center Goettingen, Goettingen, Germany; 14 Department of Interventional Neuroradiology, Hospital Vall d’Hebron, Barcelona, Spain; 15 Neuroradiology Department, CHU Caen Normandie, INSERM U1237, University Caen Normandie, Caen, France; 16 Department of Neuroradiology, University Hospital RWTH Aachen, Aachen, Germany; 17 Department of Neurology and Comprehensive Stroke Center, David Geffen School of Medicine, University of California, Los Angeles, USA; 18 Department of Clinical Research, University of Bern, Bern, Switzerland; 19 Diagnostic and Interventional Neuroradiology, CIC-IT 1415, CHRU de Tours, Tours, France; 20 Le Studium Loire Valley Institute for Advanced Studies, Orléans, France

**Keywords:** Susceptibility vessel sign, Intravenous alteplase, Thrombectomy, Ischemic stroke, SWIFT-DIRECT

## Abstract

**Background:**

The susceptibility vessel sign (SVS) on baseline MRI in acute ischemic stroke patients has been associated with better outcomes post-thrombectomy. This study aimed to investigate whether the presence of the SVS modifies the treatment effect of intravenous thrombolysis plus endovascular thrombectomy (IVT + EVT) versus thrombectomy alone (EVT alone).

**Methods:**

In this secondary analysis of the SWIFT DIRECT trial, comparing IVT + EVT versus EVT alone, treatment effect and its heterogeneity were assessed with rates of pre-interventional reperfusion (eTICI 2a–3) and successful post-interventional reperfusion (eTICI of 2b–3) according to the SVS status using adjusted multivariable logistic regression. Secondary objectives were to analyze whether the presence of SVS or its individual characteristics (location, length, width, overestimation ratio, two-layered sign) were associated with outcomes.

**Results:**

197 of the initial 408 trial participants were included in this secondary analysis, of which 52% received IVT + EVT. SVS was present in 92% of the participants (*n* = 181). There was no evidence for treatment effect heterogeneity regarding the post-interventional radiological and clinical effects of IVT + EVT versus EVT alone with strata of SVS. In SVS+ participants, IVT favored pre-interventional reperfusion (aOR 7.95, 95% CI 1.42–44.46), whereas in SVS-patients, it did not (*P* for interaction = 0.02). The individual SVS characteristics showed no significant associations with outcomes.

**Conclusion:**

Presence of SVS does not seem to modify the effect of IVT + EVT versus EVT alone. In SVS+ patients, IVT might improve pre-interventional reperfusion. There is insufficient evidence to recommend using SVS to inform IVT decisions prior to EVT.

**Supplementary Information:**

The online version of this article (10.1007/s00062-025-01501-y) contains supplementary material, which is available to authorized users.

## Introduction

The susceptibility vessel sign (SVS) seen on magnetic resonance imaging (MRI) of the brain in patients with acute ischemic stroke is the in situ visualization of the thrombus occluding the affected intracranial vessel [1]. Presence of the SVS (SVS+) as a hypointense signal in T2∗ gradient recalled echo imaging (T2∗ GRE) or in susceptibility-weighted imaging (SWI) in brain MRI corresponds to red blood cell (RBC)-rich thrombus. It is observed in 70–90% of people with stroke with visible vessel occlusion on MR-angiography [1–3].

The available evidence points toward a beneficial association of SVS+ with clinical outcomes and recanalization grades after thrombectomy [4, 5]. In contrast, a negative association of SVS+ with clinical outcome in patients treated with intravenous alteplase alone has been demonstrated [4, 6]. The influence of SVS status on the effect of intravenous alteplase plus thrombectomy (IVT + EVT) versus thrombectomy alone (EVT alone) is unclear. Identifying subgroups of people who respond differently to additional intravenous alteplase in a setting where thrombectomy is immediately available is essential for decision-making and offering the most effective acute treatment to people with stroke [7].

The SWIFT DIRECT trial was one of six international trials that aimed to evaluate the treatment effect of EVT alone compared to the combination of IVT + EVT in people with large-vessel occlusion in the anterior circulation [8]. This secondary analysis of the SWIFT DIRECT trial aimed to investigate whether SVS status modifies the treatment effect of IVT + EVT as compared with EVT alone in people with acute ischemic stroke resulting from large-vessel occlusion in the anterior circulation. Furthermore, we analyzed the effect of individual SVS characteristics on successful reperfusion as well as other secondary outcomes after treatment.

## Methods

The SWIFT DIRECT trial was a prospective randomized controlled trial for which 408 patients were randomized between November 29, 2017 and May 07, 2021. This secondary analysis was conducted according to the SWIFT DIRECT trial protocol and its corresponding revisions, which were approved by central and local ethics committees and research boards. Participants or their legal representatives had to provide written informed consent. In certain countries delayed informed consent after inclusion granted by an independent physician in emergency circumstances was used. The SWIFT DIRECT trial followed the Consolidated Standards of Reporting Trials (CONSORT) guidelines.

### Study Design and Patients

The SWIFT DIRECT trial randomized people with acute ischemic stroke resulting from large-vessel occlusion in the anterior circulation to EVT alone or IVT + EVT. Participants enrolled were eligible for thrombolysis with alteplase within 4.5 h after last known well, and for thrombectomy. The study was conducted at 48 tertiary stroke centers in Europe and Canada with thrombectomy availability 24 h a day. Inclusion criteria for SWIFT DIRECT were 1) occlusion of the intracranial internal carotid artery (ICA), the first segment (M1) of the middle cerebral artery (MCA), or both, diagnosed on admission CT angiography or MR angiography and 2) eligibility to receive alteplase within 4.5 h after last known well. Some patients were initially included with an M1 occlusion in the trial and were subsequently identified as having an occlusion of the second segment (M2) of the MCA and analyzed as such in the present study. Patients with extremely severe neurological deficits (National Institutes of Health Stroke Scale [NIHSS] 30 or more), early signs of severe tissue loss defined as Alberta Stroke Program Early CT Score (ASPECTS) of 3 or less at admission, advanced dementia, or known pre-existing disabilities (modified Rankin scale [mRS] score of ≥ 2) were excluded. This secondary analysis included only patients whose post-interventional reperfusion grade was known and who had adequate SWI quality for assessment of SVS status, as judged by a centralized imaging core laboratory.

### Imaging Analysis

The SWIFT DIRECT protocol did not stipulate whether CT or MRI should be preferred for admission imaging. All participants included in this secondary analysis received baseline MRI of the brain with T2*- or susceptibility weighted imaging (SWI) according to the enrolling center’s standard imaging protocols. MRI imaging analyses were performed by a centralized imaging core laboratory. All raters were blinded to group allocation and clinical characteristics.

**Fig. 1:**
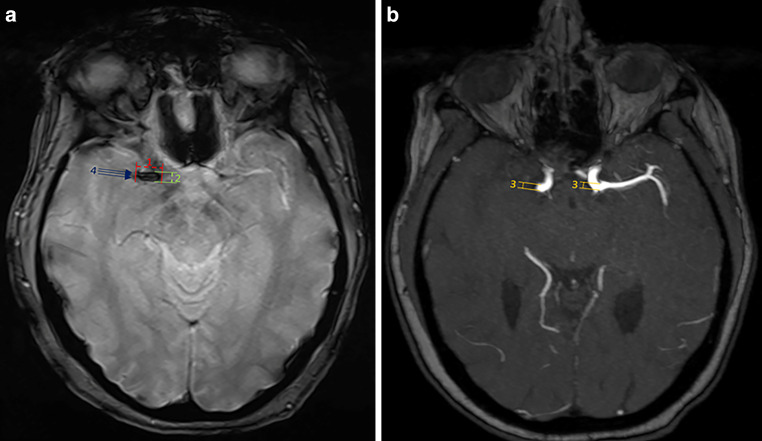
*Assessment of the susceptibility vessel sign (SVS). *Baseline MRI at admission: Axial slices of a T2*-GRE (A) and an arterial time-of-light (ToF)-sequence (B) illustrate a right-sided M1-occlusion with a positive SVS. The following assessments were performed: Length of SVS (1); Diameter of SVS (2); Overestimation Ratio: [(Diameter of SVS (2)) / (Diameter of the vessel directly proximal to the occlusion site (3) OR of the contralateral corresponding vessel)]; Presence of a 2-layered SVS: Positive if a low-intensity signal core on the T2*/SWI-sequence is surrounded by higher-intensity signal (4)

SVS was defined as the presence of a hypointense signal on T2*-weighted sequences, using either GRE or SWI, corresponding to an occluded and symptomatic intracranial artery on the MRI obtained at admission (Fig. 1). To be considered SVS+, alternative explanations for the signal hypointensity (i.e., petechial hemorrhage, neighboring vein, or microcalcification in the neighboring area) had to be excluded. SVS− was identified when a symptomatic vessel occlusion was seen on the MR angiography without a hypointense signal on T2*-GRE/SWI at the corresponding occlusion site.

For SVS+ patients, the following variables were evaluated (Fig. 1): (1) SVS localization, corresponding to the most proximal location of the SVS, specified into laterality and ICA, M1, or M2. (2) Thrombus length from the proximal to the distal end of the blooming artifact [9, 10], and (3) largest diameter of the thrombus were measured on an axial T2*-GRE/SWI-sequence and recorded in millimeters on a continuous scale. (4) Extent of the blooming artifact was evaluated using the overestimation ratio, which can be calculated by dividing the width of the SVS by the width of the occluded vessel [11]. The width of the occluded vessel was measured on MR-angiography (arterial time-of-flight (ToF)- or carotid-angiography) either directly proximal to the thrombus, or using the corresponding contralateral arterial diameter. Furthermore, (5) presence of a two-layered SVS, defined by Yamamoto et al. [12] as a low-intensity core surrounded by a higher intensity signal, was assessed.

The Horos viewer (free and open source code software (FOSS) program, distributed free of charge under the LGPL license at Horosproject.org, sponsored by Nimble Co LLC d/b/a Purview in Annapolis, MD USA) was utilized for imaging viewing and quantifications.

Reperfusion before thrombectomy and reperfusion after thrombectomy were adjudicated by the independent imaging core laboratory of the SWIFT DIRECT trial using the expanded Thrombolysis in Cerebral Infarction score (eTICI) on digital subtraction angiography (DSA) images [13].

### Outcomes

The primary outcome of this study was successful reperfusion defined as a post-interventional eTICI of 2b–3. Secondary outcomes were: modified first pass success, i.e. post-interventional eTICI2b–3 with one maneuver (only patients who effectively underwent thrombectomy were included in this analysis), pre-interventional reperfusion defined as pre-interventional eTICI 2a–3, functional independence at the 90-day visit (mRS 0–2) and mRS at the 90-day visit (shift analysis). Patients who died were assigned a mRS of 6. Safety outcomes were mortality at the 90-day visit and any intracranial hemorrhage (ICH) up to the post-randomization visit (24 ± 6 h) evaluated by the imaging core lab.

### Statistical Analysis

The primary objective was to analyze whether the presence of SVS modifies the effect of IVT + EVT versus EVT alone on successful reperfusion and secondary outcomes delineated above. The secondary objectives were to analyze (1) whether the presence of SVS is associated with successful reperfusion and secondary outcomes, and (2) whether individual SVS characteristics were associated with successful reperfusion and secondary outcomes in the SVS+ subgroup.

Differences in baseline and intervention characteristics and outcomes were compared between groups with SVS+ and SVS− at baseline using absolute and relative frequency for categorical variables, and median and interquartile range (IQR) for continuous variables. Crude comparisons were made using the Fisher’s exact test for categorical variables and the Mann-Whitney-Wilcoxon test for continuous and ordinal variables.

Treatment effect heterogeneity of intravenous alteplase plus thrombectomy versus thrombectomy alone by SVS was analyzed using regression models. Adjustments were made using two models: (1) Main model adjusting for sex and the binary stratification variables from randomization: NIHSS at baseline (≤ 17 versus >17), age (< 70 versus, ≥ 70 years), occlusion location (M1 or M2 segment of the MCA versus ICA), tandem lesions and ASPECTS (4–7 versus 8–10). For binary outcomes, we used Firth logistic regression with a penalized maximum likelihood method that reduces small-sample bias [14, 15]. (2) A simplified model to minimize the risk of overfitting including only sex, treatment allocation and age (as a continuous variable). Effects were reported as adjusted odds ratio (aOR) of IVT + EVT versus EVT alone in both SVS subgroup with 95% confidence interval (CI). Shift in mRS was analyzed using ordinal logistic regression and effects are reported as aOR for a better outcome (lower mRS) for IVT + EVT versus EVT alone in both SVS subgroups with 95% CI. Treatment effect heterogeneity according to SVS status was assessed using a Wald-test of the interaction term and *P*-values for interaction are reported. The associations of SVS status and SVS characteristics with primary and secondary outcomes were analyzed using the same adjusted regression models.

All analyses were done in Stata version 18.0. Figures were drawn with R v4.4.1 (2024-06-14).

## Results

### Study Population

**Fig. 2:**
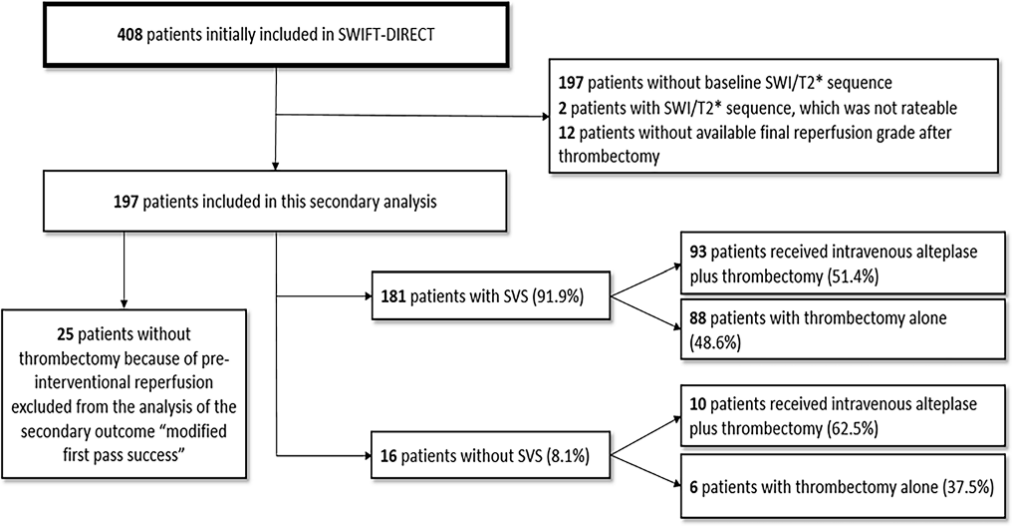
*Study flowchart*. *MRI* magnetic resonance imaging, *SVS* susceptibility vessel sign

The process for inclusion in this secondary analysis and overview of treatment allocation according to the groups (SVS+ and SVS−) are summarized in the study flowchart (Fig. 2). Of the 408 participants in the initial trial, 197 were included in this secondary analysis. Of these, 103 received IVT + EVT (52%). SVS+ was reported on baseline imaging in 181 participants (92%) and SVS− in the remaining 16 participants (8%). Of the participants with SVS+, 51% (*n* = 93/181) were allocated to IVT + EVT and of the participants with SVS−, 63% (*n* = 10/16). The treatment allocation did not differ between the two groups (*P* *=* 0.44, Table 1).

**Table 1:** Baseline characteristics by presence or absence of susceptibility vessel sign (SVS+ vs. SVS−)

	Total (*N* = 197)	SVS− (*N* = 16)	SVS+ (*N* = 181)	*P*-value
**Group—no. (%)**				
Thrombectomy alone	94 (48)	6 (38)	88 (49)	0.44
Alteplase plus thrombectomy	103 (52)	10 (63)	93 (51)	
**Age at inclusion—median (IQR)**	72 (63, 81)	61 (46, 70)	73 (65, 81)	0.002
**Female sex—no. (%)**	103 (52)	6 (38)	97 (54)	0.30
**NIHSS—median (IQR)**	16 (12, 20)	15 (8.5, 18)	16 (12, 20)	0.16
**Pre-stroke mRS—no (%)**				
0	169 (86)	13 (81)	156 (86)	0.71
1	28 (14)	3 (19)	25 (14)	
**Weight (kg)—median (IQR)**	75 (65, 85)	74 (69, 84)	75 (65, 85)	0.94
**Systolic blood pressure (mm** **Hg)—median (IQR)**	142 (126, 160)	140 (126, 149)	143 (128, 160)	0.36
**Diastolic blood pressure (mm** **Hg)—median (IQR)**	79 (70, 90)	76 (71, 92)	79 (69, 90)	0.90
**Heart rate (beats per minute)—median (IQR)**	73 (63, 86)	71 (64, 82)	73 (63, 87)	0.58
**Stroke aetiology—no. (%)**				
Large-artery atherosclerosis	32 (16)	4 (25)	28 (16)	0.27
Cardioembolism	77 (39)	3 (19)	74 (41)	
Other determined aetiology	14 (7)	1 (6)	13 (7)	
Undetermined aetiology	74 (38)	8 (50)	66 (36)	
**Risk factors—no. (%)**				
Previous ischemic stroke	23 (12)	2 (13)	21 (12)	1.00
Previous transient ischemic attack	11 (6)	2 (13)	9 (5)	0.23
History of hypertension	115 (59)	11 (69)	104 (58)	0.60
History of atrial fibrillation	18 (9)	0 (0)	18 (10)	0.37
History of hypercholesterolemia	59 (31)	4 (25)	55 (31)	0.78
Previous intracerebral hemorrhage	1 (1)	1 (6)	0 (0)	0.08
Prior myocardial infarction	19 (10)	1 (6)	18 (10)	1.00
**Medication—no. (%)**				
Warfarin or other anticoagulant	5 (3)	0 (0)	5 (3)	1.00
Aspirin	54 (27)	6 (38)	48 (27)	0.38
Statine or other lipid lowering agent	61 (31)	5 (31)	56 (31)	1.00
**Lab values—median (IQR)**				
Blood glucose level (mmol/L)	6.7 (5.8, 7.7)	5.7 (5.1, 10)	6.7 (5.9, 7.6)	0.42
International normalized ratio (INR)	1.0 (1.0, 1.1)	1.0 (1.0, 1.1)	1.0 (1.0, 1.1)	0.38
Platelet count × 10 E9 (G/L)	228 (190, 274)	249 (209, 318)	226 (189, 273)	0.18
Hemoglobin (g/L)	137 (127, 145)	138 (131, 148)	136 (127, 145)	0.59
Glomerular filtration rate (mL/min)	78 (62, 90)	88 (73, 100)	77 (62, 90)	0.07
**Imaging**				
*Baseline imaging—no. (%)*				
MRI	195 (99)	16 (100)	179 (99)	1.00
Both	2 (1)	0 (0)	2 (1)	
*ASPECTS (core lab)—median (IQR)*	8 (6, 9)	8 (7, 9)	8 (6, 9)	0.34
*ASPECTS >* *7—no. (%)*	103 (52)	12 (75)	91 (50)	0.07
*Baseline MRA occlusion site—no. (%)*				
ICA, I	7 (4)	3 (20)	4 (2)	0.06
ICA, T/L	35 (18)	4 (27)	31 (17)	
M1 proximal	75 (38)	4 (27)	71 (39)	
M1 distal	64 (33)	3 (20)	61 (33)	
M1 post-bifurcational	8 (4)	1 (6)	7 (4)	
M2 proximal superior branch	4 (2)	0 (0)	4 (2)	
M2 proximal inferior branch	2 (1)	0 (0)	2 (1)	
*Occlusion location (ICA)—no. (%)*	42 (21)	7 (44)	35 (19)	0.05
*Tandem lesion—no. (%)*	25 (13)	7 (44)	18 (10)	0.001
**Timelines—median (IQR)**				
Stroke to randomization (min)	145 (118, 182)	167 (133, 182)	144 (117, 182)	0.21
Stroke onset to imaging (min)	113 (85, 146)	134 (113, 146)	111 (84, 146)	0.21
Arrival to IV t‑PA (min)	67 (51, 80)	80 (68, 138)	65 (50, 79)	0.03
Arrival to groin puncture (min)	85 (73, 102)	104 (85, 123)	84 (73, 100)	0.002
Randomization to groin puncture (min)	29 (20, 38)	31 (22, 41)	29 (20, 38)	0.64
IV t‑PA to groin puncture (min)	24 (15, 35)	24 (7.0, 30)	24 (15, 35)	0.58

### Baseline Characteristics and Crude Comparison of the Outcomes

Median age at inclusion was 73 (IQR 65–81) and 61 (IQR 46–70) years in patients with SVS+ and SVS−, respectively (*P* *=* 0.002). In terms of other demographics (sex, weight, and blood pressure) or known risk factors, we found no evidence for any differences between patients with SVS+ and those with SVS− (Table 1). 81% of the participants with SVS+ demonstrated occlusion of the MCA (*n* = 146/181) while 44% (*n* = 7/16, *P* *=* 0.06) of those with SVS− showed an occlusion of the distal ICA. Tandem occlusion was found in 10% of participants with SVS+ (*n* = 18/181) and in 44% of those with SVS− (*n* = 7/16, *P* *=* 0.001). Compared to participants with SVS−, those with SVS+ had a shorter time from arrival at emergency department to IVT (65 min [IQR 50–79] versus 80 min [IQR 68–138], *P* *=* 0.03) and time from arrival to groin puncture (84 min [IQR 73–100] versus 104 min [IQR 85–123], *P* *=* 0.002), while time from IVT to groin puncture did not differ significantly (24 min [15–35] versus 24 min [7–30], *P* *=* 0.58).

Pre-interventional eTICI 2a–3 was observed in 7% (*n* = 13/181) of the participants with SVS+ and in 19% of patients with SVS− (*n* = 3/16, *P* *=* 0.13). Thrombectomy was performed in 92% of patients with SVS+ (*n* = 167/181) and in all patients with SVS− (*n* = 16/16, *P* *=* 0.61). The number of post-interventional successful reperfusions was similar between participants with SVS+ (95%, *n* = 172/181) and SVS− (88%, *n* = 14/16, *P* *=* 0.22). There were no significant differences between the two groups regarding the other secondary and safety outcomes (Supplemental Table 1). The distribution of stroke etiology did not differ significantly between the two groups (*P* *=* 0.27). Patients with SVS+ mainly demonstrated cardioembolism (41%, *n* = 74/181) and undetermined etiology (37%, *n* = 66/181). Most SVS− patients had an undetermined etiology (50%, *n* = 8/16) or large-artery atherosclerosis (25%, *n* = 4/16).

### Treatment Effect Heterogeneity

**Fig. 3:**
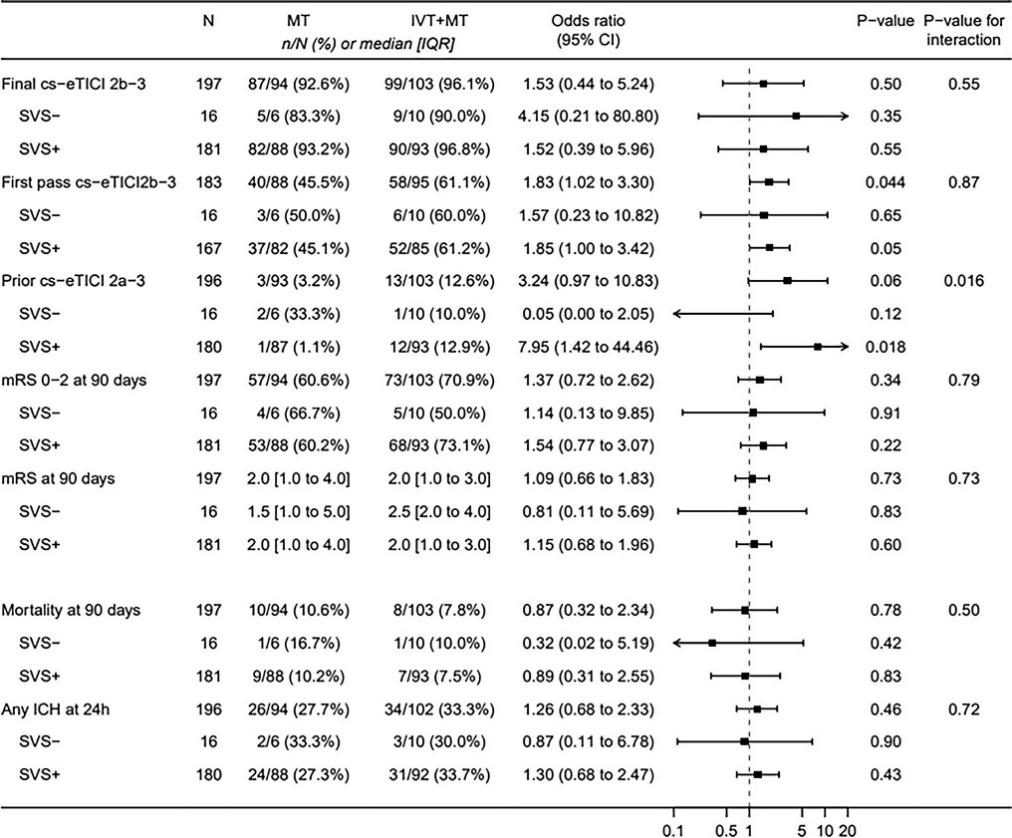
*Treatment effect for primary and secondary endpoints according to SVS status—Main model*. The effect of allocation to intravenous alteplase plus thrombectomy versus thrombectomy alone by the presence of SVS, as marginal odds ratio with 95% CI. Calculated from Firth logistic regression models adjusted for stratification factors and sex. *CI* confidence interval, *eTICI* expanded Thrombolysis in Cerebral Infarction score, *ICH* intracranial hemorrhage, *IQR* interquartile range, *IVT* intravenous thrombolysis with alteplase, *mRS* modified Rankin Scale, *MT* mechanical thrombectomy

Primary outcome—We found no evidence for any treatment effect heterogeneity regarding post-interventional successful reperfusion (*P* for interaction = 0.55, Fig. 3). There was also no evidence for higher odds of successful reperfusion in participants treated with IVT + EVT versus EVT alone overall (aOR 1.53, 95% CI 0.44–5.24) or when assessed according to SVS subgroup (SVS+ aOR 1.52, 95% CI 0.39–5.96; SVS− aOR 4.15, 95% CI 0.21–80.80). These findings remained the same with adjustment using the simplified model (Supplemental Fig. 1).

Secondary outcomes—First pass reperfusion occurred in 61% of patients in the IVT + EVT group versus 46% in the EVT alone group (aOR 1.83, 95% CI 1.02–3.30, *P* *=* 0.04, Fig. 3), however, the interaction was not significant (*P* for interaction = 0.87). There was no evidence that treatment allocation contributed to a first pass effect in SVS subgroups (SVS+ aOR 1.85, 95% CI 1.00–3.42; SVS− aOR 1.57, 95% CI 0.23–10.82).

There was some evidence for a treatment effect heterogeneity for pre-interventional reperfusion (*P* for interaction *=* *0.02*) with higher pre-interventional reperfusion after IVT + EVT in participants with SVS+ (aOR 7.95, 95% CI 1.42–44.46, *P* *=* 0.02) but not for those with SVS− (aOR 0.05, 95% CI 0.00–2.05, *P* *=* 0.12) or all participants (aOR 3.24, 95% CI 0.97–10.83, *P* *=* 0.06).

Regarding functional outcomes at 90 days (mRS 0–2 and mRS shift) and safety outcomes, we found no evidence of treatment effect heterogeneity.

In the simplified model, treatment effect heterogeneity for pre-interventional reperfusion (*P* for interaction *=* *0.02*) was significant with higher pre-interventional reperfusion after IVT + EVT for all participants (aOR 3.76, 95% CI 1.12–12.58, *P* *=* 0.03) and remained significant for patients with SVS+ (aOR 8.83, 95% CI 1.58–49.34, *P* *=* 0.01). All other results were comparable with the main model.

### Association of SVS Status with Outcomes

Post-interventional successful reperfusion was observed in 172 participants (95%) with SVS+ and 14 participants (88%) with SVS- and was associated with SVS+ (aOR 6.83, 95% CI 1.14–40.82, *P* *=* 0.04). 67% (*n* = 121/181) of the SVS+ participants compared to 56% (*n* = 9/16) of the SVS− participants reached functional independence (mRS 0–2) at 90 days (aOR 4.43, 95% CI 1.29–15.20, *P* *=* 0.02). No further association of the SVS with other secondary or safety outcomes could be seen. (Supplemental Fig. 2) These findings remained unchanged using the simplified model.

### Association of SVS Characteristics On Outcomes

SVS analysis was performed on MRI-sequences sensitive for magnetic susceptibility, using SWI in 22% (44/197), T2*-GRE in 77% (152/197), or both in 1% (1/197) of all included participants (Supplemental Table 2).

In SVS+ patients, 56% (102/181) of all SVS were localized within the right-sided anterior circulation territory. The most proximal end of the SVS was localized within the terminal ICA in 12%, within M1 in 85%, and within M2 in 3% of the cases. Median SVS length, diameter, and overestimation ratio were 12 mm (IQR 8.4–16), 4.3 mm (IQR 3.5–5.5), and 1.7 (IQR 1.3–2.1), respectively. 17% of all SVS were two-layered (31/181). (Supplemental Table 3).

No SVS characteristic showed a significant association with final successful reperfusion (Supplemental Fig. 3), or any of the secondary or safety outcomes. There was a tendency towards lower odds for pre-interventional reperfusion with increasing SVS diameter (*P* *=* 0.05, Supplemental Fig. 4), which could not be corroborated using the simplified model (*P* *=* 0.10). Tendencies towards higher odds for any ICH at 24 h could be observed with increasing SVS length (*P* *=* 0.07) and presence of a two-layered SVS (*P* *=* 0.08), and were even stronger using the simplified model (*P* *=* 0.05, *P* *=* 0.02) (Supplemental Fig. 5).

## Discussion

The main findings of this study are as follows: (1) The SVS status does not seem to modify the effect of allocation to IVT + EVT versus EVT alone neither on the rate of post-interventional successful reperfusion nor on functional outcomes. (2) IVT using alteplase may particularly favor pre-interventional reperfusion in patients with SVS+ in the setting of proximal intracranial occlusion of the anterior circulation. (3) SVS+ might be associated with higher rates of successful reperfusion and functional independence after 90 days. (4) SVS characteristics did not show any association with outcomes.

According to the current retrospective evidence on thrombectomy, compared to those with SVS−, stroke patients with SVS+ on baseline MRI demonstrated better radiological outcomes (successful reperfusion defined as a TICI score of 2b–3) and better clinical outcomes at 90 days (mRS ≤ 2) after thrombectomy [4, 6, 16]. These differences are attributed to the physical characteristics of the types of thrombi associated with SVS+ or SVS−. As mentioned in the introduction, in patients with SVS+, the proportion of RBC in the histological composition of retrieved thrombi is higher than in patients with SVS− [1]. RBC-rich thrombi are believed to be more easily retrievable because they are less rigid and more deformable than the platelet- and fibrin-rich thrombi, mostly found in patients with SVS− [17]. Platelet- and fibrin-rich thrombi are stiffer and more elastic, reducing the likelihood of engagement and interaction with the thrombectomy device [18, 19]. According to Gunning et al., platelet- and fibrin-rich clots also have a higher coefficient of friction and, therefore, a greater resistance to sliding along the inside of the vessel during clot retrieval. [20, 21] In this secondary analysis of the SWIFT DIRECT trial, the overall percentage of patients with SVS+ (92%) was higher than in available retrospective studies (70–90%) [1–3, 16, 22]. This difference could be explained by our study’s high number of cardioembolic stroke etiology and inclusion of anterior and proximal large-vessel occlusion, which are generally associated with SVS+ [23]. Comparing patients with SVS+ and SVS− in our study (Fig. 3), there was no apparent treatment effect heterogeneity between IVT + EVT and EVT alone regarding the success of the post-interventional reperfusion (*P* for interaction = 0.55), the achievement of good functional outcomes (mRS ≤ 2) at 90 days (*P* for interaction = 0.79), or the modified first pass success (*P* for interaction = 0.87). Although not often explicitly differentiated in retrospective studies, patients with distal large-vessel occlusion (M2 segment of the MCA) undergoing thrombectomy are more likely to present with SVS− than patients with occlusion of the distal ICA or M1 segment of the MCA [16]. The low number of patients with occlusion of the M2 segment of the MCA erroneously included in the SWIFT DIRECT trial could explain why patients with SVS− did not show poorer post-interventional outcomes than patients with SVS+ in this secondary analysis. Although the absence of SVS was previously associated with a shorter time between symptom-onset and imaging (expected in the context of the hyperacute design of SWIFT DIRECT trial) [24, 25], our findings showed an overall low number of SVS− (8%). However, our study demonstrated a higher prevalence of SVS− clots in the distal ICA as compared to SVS+ clots (44% vs. 19%), most likely due to location-dependent clot composition. The distal ICA is a common site for atherosclerotic plaque formation and rupture, where immediate clot formation primarily involves platelets and fibrin [26, 27]. Additionally, the hemodynamic environment with high-flow and high wall shear stress in the distal ICA promotes platelet adhesion and aggregation, leading to more dense clots rich in platelets and fibrin, whereas RBCs are more likely to be washed distally [27]. In two recent meta-analyses of the current evidence regarding response to intravenous alteplase alone, patients with SVS+ demonstrated lower reperfusion rates and poorer functional outcomes than those with SVS− [4, 6]. According to Liu et al., SVS− thrombi are more responsive to intravenous alteplase with recombinant tissue plasminogen activator because of their higher fibrin content [4]. Furthermore, because of the time dependence of oxyhaemoglobin desaturation of erythrocytes, older thrombi have higher levels of deoxyhemoglobin and hemosiderin, resulting in inhomogeneity of the magnetic field, which is what renders these thrombi more visible as SVS+ on SWI sequences [1, 28, 29]. Older thrombi are more difficult to dissolve with intravenous alteplase because they become more compressed by retraction of the thrombus (increase in density and decrease in size). This is due to platelet contraction and increased fibrin deposition (leading to smaller pores within the fibrin network, reducing the penetration of intravenous alteplase into the thrombus) [4, 30]. Additionally, leukocytes infiltrate thrombi over time and their activation leads to resistance to intravenous alteplase [30]. According to our analysis (Fig. 3), when intravenous alteplase is combined with thrombectomy, compared to SVS−, SVS+ favored pre-interventional reperfusion (*P* = 0.02). This finding was confirmed by a significant interaction of the SVS status on the association between pre-interventional reperfusion and treatment allocation (*P* for interaction *=* 0.02). Again, our results cannot be directly compared with the current evidence, as most studies intentionally included occlusions of the M2 segment of the MCA and/or other anterior and posterior occlusion sites [2, 31–33].

Furthermore, there was evidence for an association between SVS+, compared to SVS-, and post-interventional successful reperfusion (aOR 6.83, 95% CI 1.14–40.82, *P* *=* 0.04) and functional independence (mRS 0–2) at 90 days (aOR 4.43, 95% CI 1.29–15.20, *P* *=* 0.02), which is consistent with findings of previous studies [16, 34].

Regarding individual characteristics of the SVS, literature hints towards an association between SVS length and successful reperfusion, while there have been heterogeneous findings for the SVS width [2, 6, 28, 30, 35]. Although the overestimation ratio and two-layered SVS have been linked to cardioembolic stroke aetiology [36, 37], we found no studies investigating their association with successful reperfusion. In our secondary analysis of the SWIFT DIRECT trial, we could not find any evidence for an association between any individual SVS characteristic on successful reperfusion or secondary outcomes.

Based on the results presented in our study, there is currently insufficient evidence to recommend using the SVS status when deciding on the advisability of treatment with IVT + EVT versus EVT alone in stroke patients with large-vessel occlusion of the anterior circulation.

## LIMITATIONS

Firstly, we performed a post hoc analysis of a randomized clinical trial, which was not designed to answer the specific question addressed in this study. Secondly, the study population included only patients with anterior and proximal vessel occlusion (ICA, M1 and M2 segment of the MCA) who were eligible for thrombectomy. Consequently, our results cannot be generalized to patients with distal occlusion of the MCA and other anterior or posterior occlusion. Third, due to the small sample size (especially in the SVS− group) the statistical power is low and absence of evidence cannot be interpreted as evidence of absence. In addition, there is a chance of false-positive findings due to the many outcomes tested (alpha error inflation) and significant interaction findings concerning secondary endpoints should be handled cautiously. Further studies with a larger sample size addressing the same question are needed to confirm our results. Fourth, analysis of treatment effect heterogeneity across individual SVS characteristics was not feasible due to limited power for individual variables. Fifth, the use of different imaging protocols and different MRI field strengths by the individual study sites may have influenced the assessment of SVS status and subsequently the impact of the SVS status on the treatment allocated.

## Conclusion

Our study did not find any influence of the SVS status on the treatment effect after allocation to intravenous alteplase plus thrombectomy compared to thrombectomy alone regarding final reperfusion rates and functional outcomes at 90 days in stroke patients with large-vessel occlusion of the anterior circulation. In participants with SVS+, the pre-interventional reperfusion rate after intravenous alteplase was higher than in those treated with thrombectomy only. Independent of treatment allocation, there was evidence for SVS+ being associated with successful reperfusion and functional independence at 90 days. However, there is insufficient evidence to recommend using SVS for informing decisions in favor of or against intravenous alteplase before thrombectomy. Individual SVS characteristics were not associated with any clinical or imaging outcome. Further studies with a larger sample size and including real-world data are needed to confirm our findings.

## Supplementary Information

Supplemental Tables and Figures from this secondary analysis of the SWIFT DIRECT Trial.

## Data Availability

Data from the SWIFT DIRECT trial are available as a complete deidentified dataset together with a data dictionary. Reasonable requests after clearance by the local ethics committee (if applicable) can be made by sending an email together with a research plan to urs.fischer@insel.ch.

## Abbreviations

ASPECTS: Alberta Stroke Program Early CT Score
eTICI: Expanded Treatment In Cerebral Infarction
HVS: Hyperdense vessel sign
ICA: Internal carotid artery
MCA: Middle cerebral artery
mRS: Modified Rankin Scale
NIHSS: National Institutes of Health Stroke Scale
RBC: Red blood cell
SVS: Susceptibility vessel sign
SWI: Susceptibility-weighted imaging
T2*-GRE: T2*-weighted Gradient-Recalled Echo
