# Supplementary material for: Susceptibility Vessel Sign and Intravenous Alteplase in Stroke Patients Treated with Thrombectomy: A Secondary Analysis of the SWIFT DIRECT Trial
Source: Clin Neuroradiol. 2025 Feb 20;35(3):483–93. doi: 10.1007/s00062-025-01501-y (PMC12454450; doi:10.1007/s00062-025-01501-y)

**SUPPLEMENT MATERIAL**

**Susceptibility Vessel Sign and Intravenous Alteplase in Stroke Patients Treated with Thrombectomy:
A Secondary Analysis of the SWIFT DIRECT Trial**

**TABLE OF CONTENTS**

- Table S1: Descriptives for all outcomes by presence or absence of susceptibility

vessel sign (SVS+ vs SVS-)

- Table S2: Baseline characteristics by SWI type
- Table S3: Characteristics of susceptibility vessel sign (in SVS+ participants)
- Table S4: Details of thrombectomy by presence of absence of susceptibility vessel

sign (SVS+ vs SVS-)

- Figure S1: Figure S1: Treatment effect for primary and secondary endpoints

according to SVS status – Simplified model

- Figure S2: Secondary objective – Main effect
- Figure S3: Secondary objective – Association between SVS characteristics and

reperfusion success (final cs-eTICI 2b-3): Adjusted model

- Figure S4: Secondary objective – Association between SVS characteristics
   and pre-interventional reperfusion (prior cs-eTICI 2a-3): Adjusted model
- Figure S5: Secondary objective – Association between SVS characteristics and any

ICH at 24h: Small adjusted model

- Figure S6: The mRS shift at 90 days stratified by susceptibility vessel sign (SVS)

status

|  | **Total**  (N = 197) | **SVS-**  (N = 16) | **SVS+**  (N = 181) | **P-value** |
| --- | --- | --- | --- | --- |
| Final cs-eTICI 2b-3 – no. (%)  First pass cs-eTICI 2b-3 – no. (%)  Prior cs-eTICI 2a-3 – no. (%)  mRS 0-2 at 90 days – no. (%)  mRS at 90 days – no. (%)  0  1  2  3  4  5  6  Median (IQR)  Mortality at 90 days – no. (%)  Any ICH at 24h – no. (%) | 186 (94)  98 (54)  16 (8)  130 (66)  27 (14)  57 (29)  46 (23)  27 (14)  17 (8)  5 (3)  18 (9)  2 (1, 3)  18 (9)  60 (31) | 14 (88)  9 (56)  3 (19)  9 (56)  3 (19)  2 (13)  4 (25)  2 (13)  2 (12)  1 (6)  2 (12)  2 (1, 4)  2 (13)  5 (31) | 172 (95)  89 (53)  13 (7)  121 (67)  24 (13)  55 (31)  42 (23)  25 (14)  15 (8)  4 (2)  16 (9)  2 (1, 3)  16 (9)  55 (31) | 0.22  1.00  0.13  0.42  0.50  0.42  0.64  1.00 |

**SUPPLEMENTAL TABLES**

**Table S1:** Descriptives for all outcomes by presence or absence of susceptibility vessel sign (SVS+ vs SVS-)

|  | **Total**  (N = 197) | **SWI**  (N = 44) | **T2***  (N = 152) | **SWI+T2***  (N = 1) | **P-value** |
| --- | --- | --- | --- | --- | --- |
| Group – no. (%)  Thrombectomy alone  Alteplase plus thrombectomy | 94 (48)  103 (52) | 21 (48)  23 (52) | 72 (47)  80 (53) | 1 (100)  0 (0) | 0.79 |
| Age at inclusion – median (IQR) | 72 (63, 81) | 72 (64, 82) | 72 (61, 80) | 66 (66, 66) | 0.71 |
| Female sex – no. (%) | 103 (52) | 23 (52) | 80 (53) | 0 (0) | 0.79 |
| NIHSS – median (IQR) | 16 (12, 20) | 15 (10, 19) | 17 (12, 20) | 15 (15, 15) | 0.42 |
| Pre-stroke mRS – no (%)  0  1 | 169 (86)  28 (14) | 38 (86)  6 (14) | 130 (86)  22 (14) | 1 (100)  0 (0) | 1.00 |
| Weight (kg) – median (IQR) | 75 (65, 85) | 70 (65, 80) | 76 (66, 86) | 75 (75, 75) | 0.31 |
| Systolic blood pressure (mmHg) – median (IQR) | 142 (126, 160) | 152 (130, 168) | 140 (125, 157) | 132 (132, 132) | 0.12 |
| Diastolic blood pressure (mmHg) – median (IQR) | 79 (70, 90) | 80 (71, 91) | 77 (70, 90) | 67 (67, 67) | 0.42 |
| Heart rate (beats per minute) – median (IQR) | 73 (63, 86) | 78 (67, 91) | 72 (63, 85) | 67 (67, 67) | 0.34 |
| Stroke aetiology – no. (%)  Large-artery atherosclerosis  Cardioembolism  Other determined aetiology  Undetermined aetiology | 32 (16)  77 (39)  14 (7)  74 (38) | 7 (16)  18 (41)  4 (9)  15 (34) | 25 (16)  59 (39)  10 (7)  58 (38) | 0 (0)  0 (0)  0 (0)  1 (100) | 0.92 |
| Risk factors – no. (%)  Previous ischemic stroke  Previous transient ischemic attack  History of hypertension  History of atrial fibrillation  History of hypercholesterolemia  Previous intracer. hemorrhage  Prior myocardial infarction | 23 (12)  11 (6)  115 (59)  18 (9)  59 (31)  1 (1)  19 (10) | 4 (10)  1 (2)  26 (62)  6 (15)  18 (42)  1 (2)  7 (17) | 19 (13)  10 (7)  89 (59)  12 (8)  40 (27)  0 (0)  12 (8) | 0 (0)  0 (0)  0 (0)  1 (100)  0 (0)  0 (0) | 0.81  0.46  0.57  0.30  0.04  0.22  0.22 |
| Medication – no. (%)  Warfarin or other anticoagulant  Aspirin  Statine/other lipid lowering agent | 5 (3)  54 (27)  61 (31) | 1 (2)  14 (32)  16 (36) | 4 (3)  40 (26)  44 (29) | 0 (0)  0 (0)  1 (100) | 1.00  0.68  0.21 |
| Lab values – median (IQR)  Blood glucose level (mmol/L)  International normalized ratio  Platelet count x 10 E9 (G/L)  Hemoglobin (g/L)  Glomerular filtr. rate (mL/min) | 6.7 (5.8, 7.7)  1.0 (1.0, 1.1)  228 (190, 274)  137 (127, 145)  78 (62, 90) | 6.3 (5.7, 7.5)  1.0 (1.0, 1.1)  211 (180, 254)  134 (128, 142)  76 (65, 88) | 6.8 (5.8, 7.8)  1.0 (1.0, 1.1)  229 (196, 280)  138 (126, 146)  79 (61, 90) | 77 (77, 77)  138 (138, 138)  90 (90, 90) | 0.29  0.02  0.03  0.39  0.51 |
| Imaging  Baseline imaging – no. (%)  MRI  both  ASPECTS – median (IQR)  ASPECTS > 7 – no. (%)  Baseline MRA occl. site– no. (%)  ICA, I  ICA, T/L  M1 proximal  M1 distal  M1 post-bifurcational  M2 proximal superior branch  M2 proximal inferior branch  Occl. location (ICA) – no. (%)  Tandem lesion – no. (%) | 195 (99)  2 (1)  8 (6, 9)  103 (52)  7 (4)  35 (18)  75 (38)  64 (33)  8 (4)  4 (2)  2 (1)  42 (21)  25 (13) | 44 (100)  0 (0)  8 (7, 9)  21 (48)  3 (7)  5 (11)  15 (34)  18 (41)  2 (5)  1 (2)  0 (0)  8 (18)  6 (14) | 150 (99)  2 (1)  8 (6, 9)  82 (54)  4 (3)  29 (19)  60 (40)  46 (31)  6 (4)  3 (2)  2 (1)  33 (22)  19 (13) | 1 (100)  0 (0)  8 (8, 8)  0 (0)  0 (0)  1 (100)  0 (0)  0 (0)  0 (0)  0 (0)  0 (0)  1 (100)  0 (0) | 1.00  0.91  0.44  0.43  0.22  0.83 |
| Timelines – median (IQR)  Stroke to randomization (min)  Stroke onset to imaging (min)  Arrival to IV t-PA (min)  Arrival to groin puncture (min)  Random. to groin puncture (min)  IV t-PA to groin puncture (min) | 145 (118, 182)  113 (85, 146)  67 (51, 80)  85 (73, 102)  29 (20, 38)  24 (15, 35) | 133 (111, 184)  100 (78, 146)  61 (47, 79)  76 (70, 92)  26 (17, 35)  20 (9, 30) | 149 (120, 182)  113 (88, 146)  68 (55, 80)  87 (75, 106)  29 (21, 39)  24 (17, 35) | 148 (148, 148)  132 (132, 132)  65 (65, 65)  22 (22, 22) | 0.35  0.34  0.31  0.005  0.24  0.11 |

**Table S2:** Baseline characteristics by SWI type.

|  | **SVS+**  (N=181) |
| --- | --- |
| Type of SWI – no. (%)  SWI  T2*  T2*+SWI  Other  None  Side of SVS – no. (%)  Left  Right  SVS Location – no. (%)  ICA  M1  M2  SVS length (mm) – median (IQR)  SVS diameter (mm) – median (IQR)  Overestimation ratio – median (IQR)  Two-layered SVS – no. (%) | 42 (23)  138 (76)  1 (1)  0 (0)  0 (0)  79 (44)  102 (56)  22 (12)  153 (85)  6 (3)  12 (8.4, 16)  4.3 (3.5, 5.5)  1.7 (1.3, 2.1)  31 (17) |

**Table S3:** Characteristics of susceptibility vessel sign (in SVS+ participants).

**Table S4:** Details of thrombectomy by presence of absence of susceptibility vessel sign (SVS+ vs SVS-)

|  | **Total**  (N = 197) | **SVS-**  (N = 16) | **SVS+**  (N = 181) | **P-value** |
| --- | --- | --- | --- | --- |
| Number of passes – median (IQR)  Any mechanical device used – no. (%)  Mechanical thrombectomy performed – no. (%)  Balloon guide catheter used – no. (%)  Distal aspiration catheter used – no (%)  Extracranial Stenting – no. (%)  Peri-Interventional Aspirin – no. (%)  Further thrombectomy device used after Solitaire – no. (%)  Conscious sedation – no. (%)  General anesthesia – no. (%)  Reason for general anesthesia – no. (%)  Hospital standard practice  Clinically indicated | 1 (1, 2)  192 (98)  183 (93)  80 (41)  153 (78)  14 (7)  20 (10)  62 (32)  134 (68)  57 (29)  41 (72)  16 (28) | 1 (1, 3)  16 (100)  16 (100)  9 (56)  11 (69)  3 (19)  4 (25)  5 (31)  11 (69)  4 (25)  3 (75)  1 (25) | 1 (1, 2)  176 (97)  167 (92)  71 (39)  142 (79)  11 (6)  16 (9)  57 (32)  123 (68)  53 (29)  38 (72)  15 (28) | 0.65  1.00  0.61  0.20  0.36  0.09  0.06  1.00  1.00  1.00  1.00 |

**SUPPLEMENTAL FIGURES**

**Figure S1: Treatment effect for primary and secondary endpoints according to SVS status – Simplified model**

The effect of allocation to intravenous alteplase plus thrombectomy versus thrombectomy alone by the presence of SVS, as marginal odds ratio with 95% CI. Calculated from Firth logistic regression models adjusted for age (assuming a linear effect) and sex.


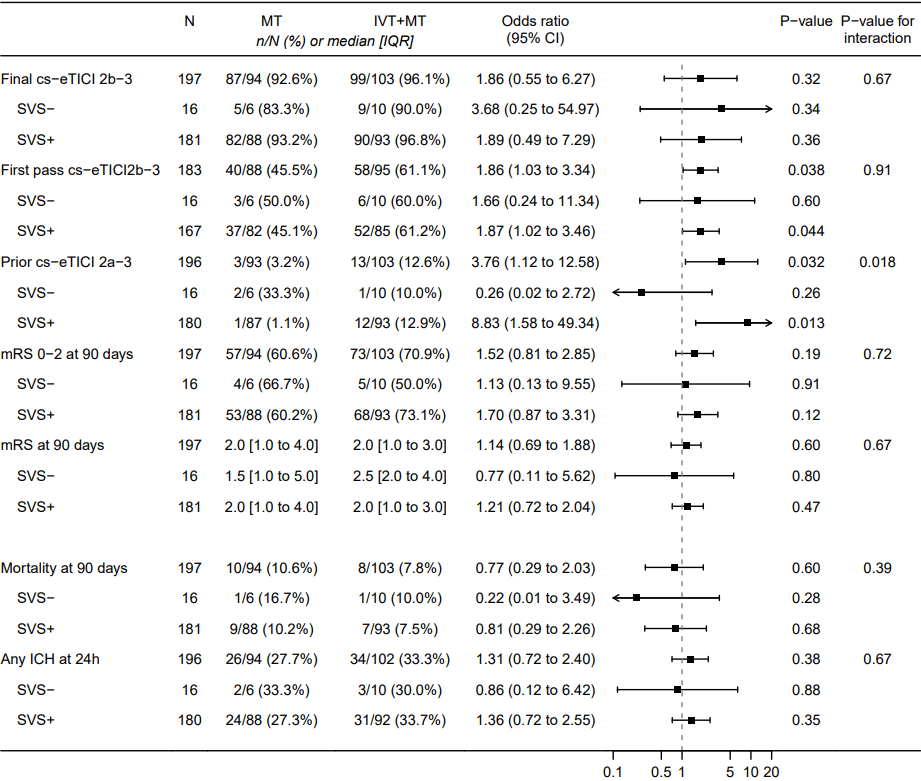

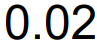

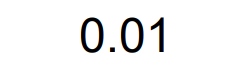

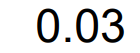

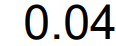

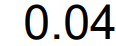


**Figure S2: Secondary objective – Main effect**

Association between susceptibility vessel sign (SVS+ vs SVS-) and outcomes as marginal odds ratio with 95% confidence interval (CI). Calculated from Firth logistic regression models adjusted for the stratification factors (assuming a linear effect if continuous), allocation and sex.


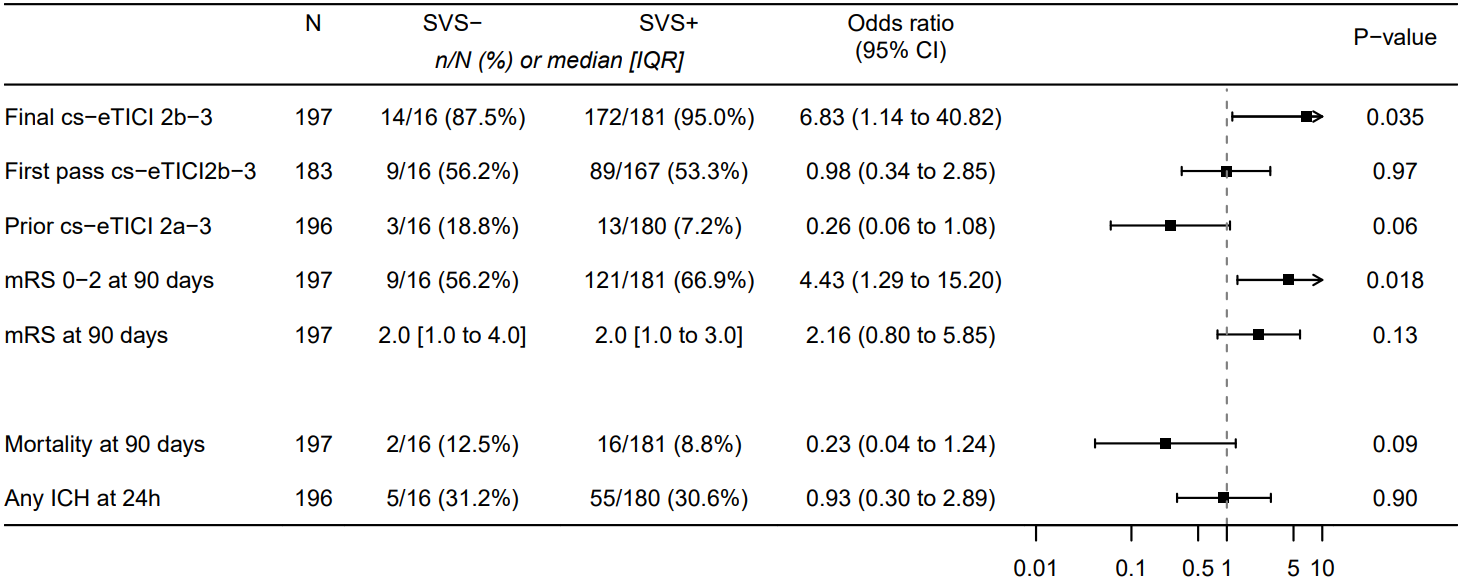

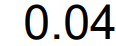

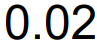


**Figure S3: Secondary objective – SVS characteristics: Adjusted model**

Association between SVS characteristics and reperfusion success (final cs-eTICI 2b-3), as marginal odds ratio with 95% confidence interval (CI). Calculated from Firth logistic regression models adjusted for the stratification factors (dichotomized if continuous), allocation and sex.


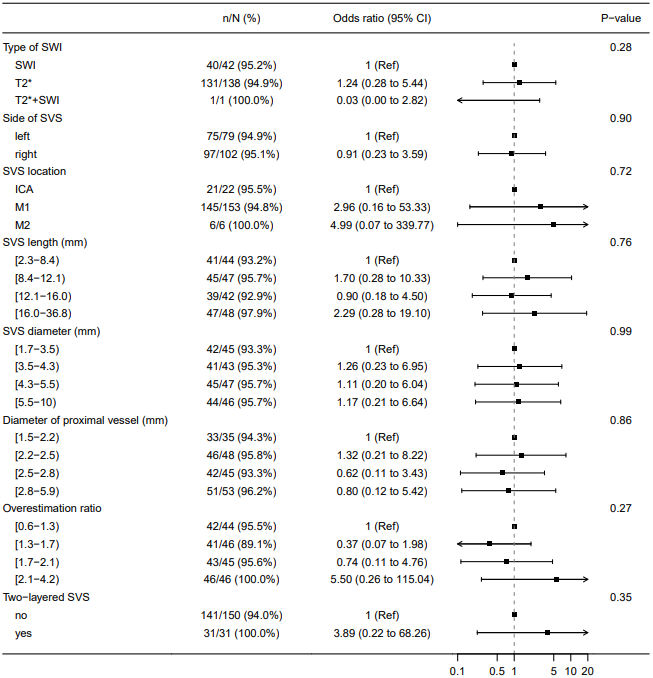


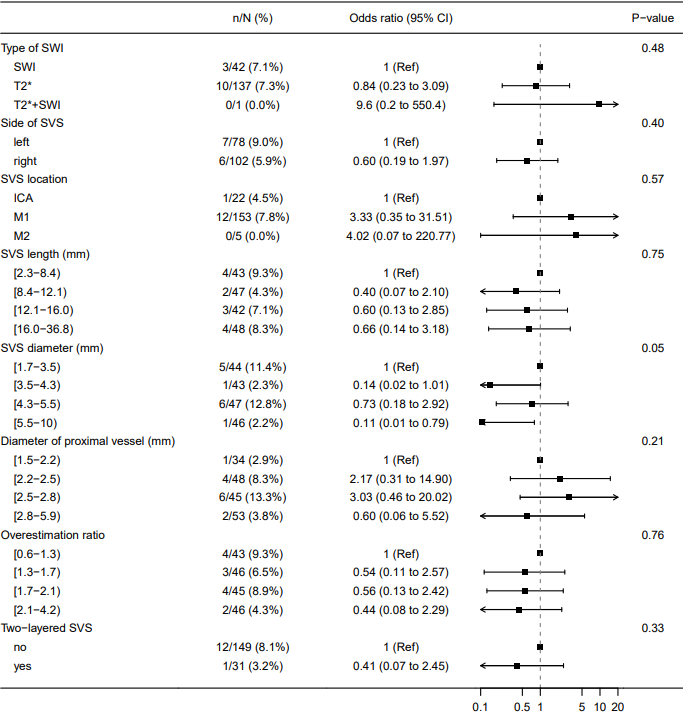


**Figure S4: Secondary objective – SVS characteristics: Adjusted model**

Association between SVS characteristics and pre-interventional reperfusion (prior cs-eTICI 2a-3), as marginal odds ratio with 95% confidence interval (CI). Calculated from Firth logistic regression models adjusted for the stratification factors (dichotomized if continuous), allocation and sex.

**Figure S5: Secondary objective – Effect of SVS characteristics: Small adjusted model**

Association between SVS characteristics and any ICH at 24h, as marginal odds ratio with 95% confidence interval (CI). Calculated from Firth logistic regression models adjusted for age (assuming a linear

effect), allocation and sex.


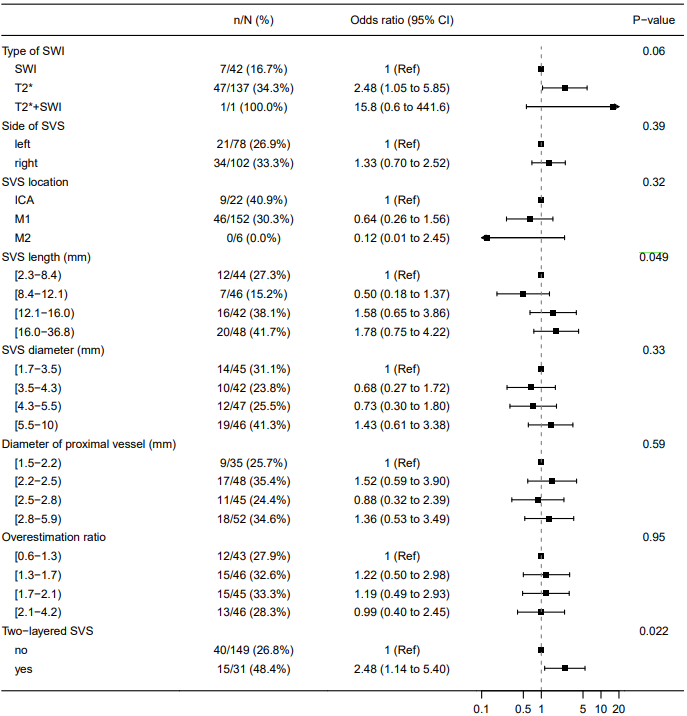

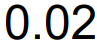

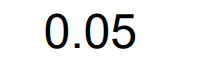


**Figure S6:** The mRS shift at 90 days stratified by susceptibility vessel sign (SVS) status


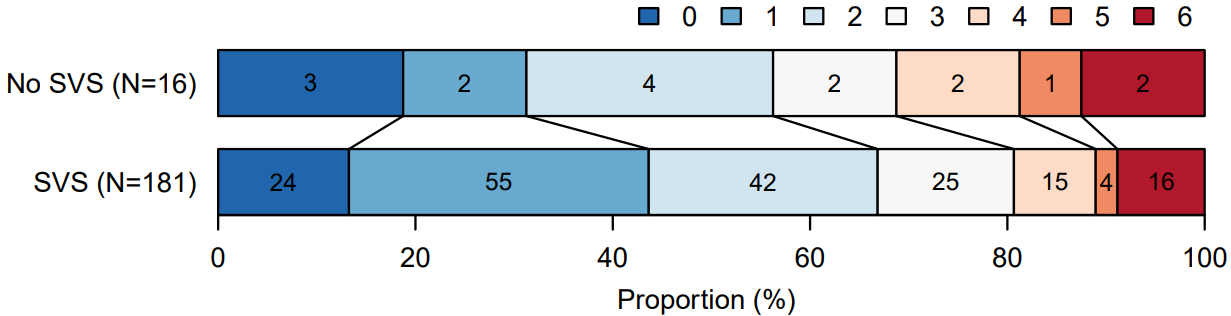

Supplement: Supplementary file 1 — Supplemental Tables and Figures from this secondary analysis of the SWIFT DIRECT Trial. [file 62_2025_1501_MOESM1_ESM.docx]
